# Supplementary material for: Personalized antibiograms for machine learning driven antibiotic selection
Source: Commun Med (Lond). 2022 Apr 8;2:38. doi: 10.1038/s43856-022-00094-8 (PMC9053259; doi:10.1038/s43856-022-00094-8)
Supplement: Supplementary file 3 — Supplemental Material [file 43856_2022_94_MOESM3_ESM.pdf]

# Supplementary Materials

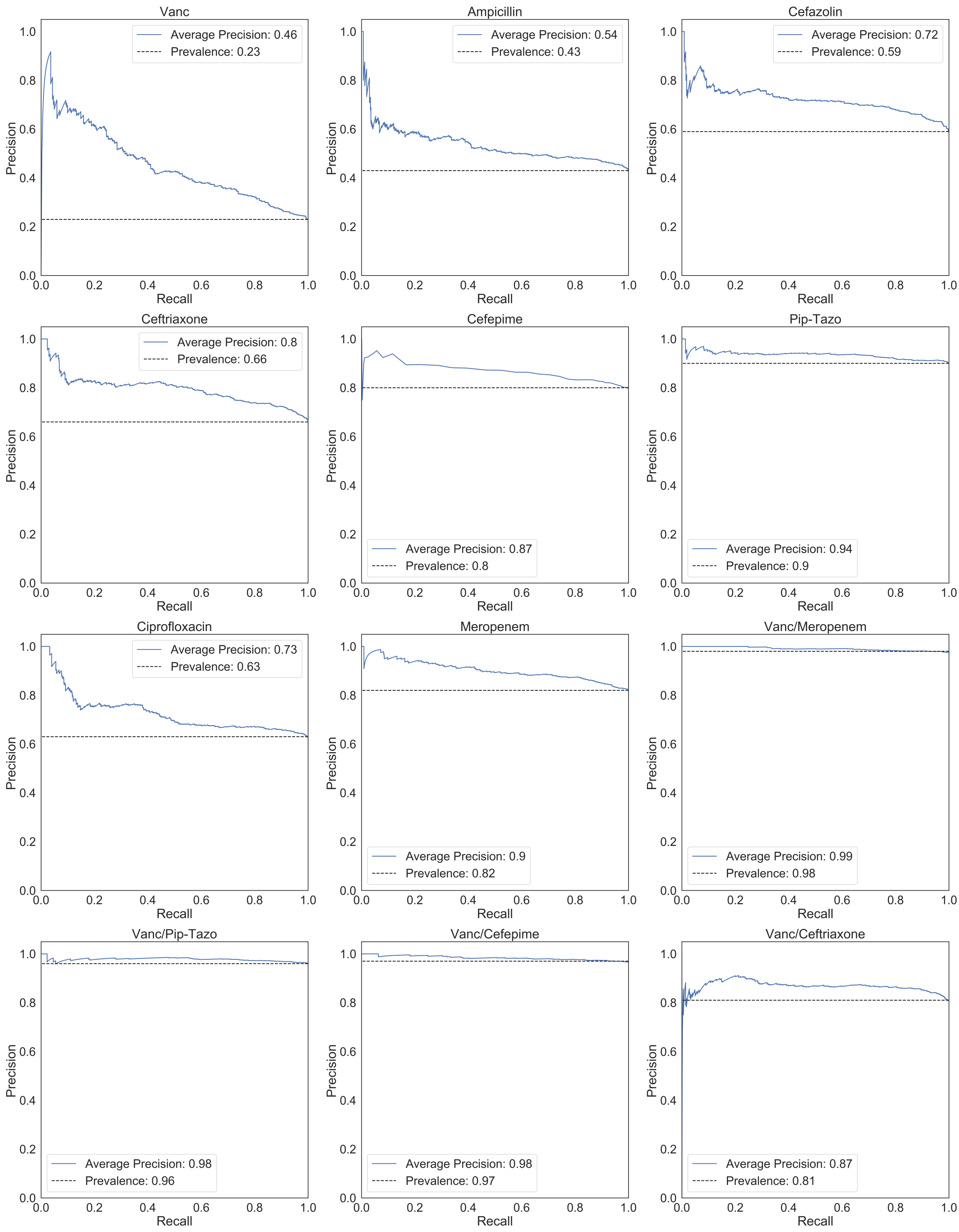


Supplementary Figure 1: Precision recall curves for each of the twelve personalized antibiogram models trained using the Stanford Dataset. The y-axis (precision) is the proportion of observations with a positive label conditioned on the model predicting a positive label. The x-axis (recall) is the proportion of total positive observations predicted as positive. These measures are calculated at varying probability thresholds. For each plot, we list the average precision and positive class prevalence which is baseline performance.

## Supplementary Note 1

In Supplementary Table 1 we show the results of our model selection procedure. We report AUROC on the validation set for each of the finalists for each model class. We searched over a grid ($10^{-8}$ to $10^{8}$ in powers of 10) of regularization hyperparameters for the lasso and ridge regressions. The lasso used the liblinear solver, the ridge logistic regression used the lbfgs solver. For the random forest, the number of trees were set to 1,000, and we swept over min sample splits (2, 10, 50, 100) and max features ('sqrt', 'log2', None). For the gradient boosted tree, we swept over learning rates (0.01, 0.05, 0.1, 0.5) and the number of base leaves (2, 8, 16, 32, 64). The max number of boosting rounds was set to 1,000 and we used early stopping with a tolerance of 10.

## Supplementary Note 2

The following ICD codes were used in the definition the electronic phenotype that flagged patients with negative microbial cultures that lacked bacterial infection. If any of the following codes were associated with the admission in question, the electronic phenotype did not flag as lacking an infection.

- ICD9 995.92 Sepsis
- ICD9 995.92 Severe Sepsis
- ICD9 481 Pneumococcal pneumonia
- ICD9 482 Other bacterial pneumonia
- ICD9 483 Pneumonia due to other specified organism
- ICD9 484 Pneumonia in infectious diseases classified elsewhere
- ICD9 485 Bronchopneumonia org NOS
- ICD9 486 Pneumonia, organism NOS
- ICD9 590 Infections of kidney
- ICD10 A41 Other sepsis
- ICD10 J13 Pneumonia due to Streptococcus pneumoniae
- ICD10 J15 Bacterial pneumonia, not elsewhere classified
- ICD10 J16 Pneumonia due to other infectious organisms, not elsewhere classified
- ICD10 J17 Pneumonia in diseases classified elsewhere
- ICD10 J18 Pneumonia, unspecified organism
- ICD10 N10 Acute pyelonephritis
- ICD10 N11 Chronic tubulo-interstitial nephritis
- ICD10 N12 Tubulo-interstitial nephritis, not specified as acute or chronic
- ICD10 N39.0 Urinary tract infection, site not specified
- ICD10 J06 Acute upper respiratory infections of multiple and unspecified sites
- ICD10 A49 Bacterial infection of unspecified site
- ICD10 J22 Unspecified acute lower respiratory infection
- ICD10 R65.2 Severe sepsis (with and without septic shock)

## Supplementary Note 3

In this section we describe two additional experiments conducted to demonstrate how well the linear programming based antibiotic allocation procedure performs when using gram stain and species specific susceptibility probabilities (probabilities that resemble those in normal antibiograms). We note that although at decision time (time of empiric antibiotic selection) gram stain and species identity is unknown, these analyses demonstrate how well our decision support could work in the advent of rapid diagnostic technology.

We performed the linear programming antibiotic allocation procedure under two conditions.

- Condition 1: The gram stain is known at the time of the antibiotic recommendation.
- Condition 2: The species identity is known at the time of the antibiotic recommendation.

In both experiments we feed summary measures of antibiotic susceptibilities into the linear programming procedure. When these summary measures are species specific (condition 2), they are directly comparable to normal antibiogram values.

In both conditions the linear programming formulation is identical to the formulation used in the main text.

The coverage rate of the linear programming based optimizer in condition 1 was 89.4% — 95% CI [87.1%, 91.4%]. The coverage rate of the linear programming based optimizer in condition 2 (organism species is known) was 94.8% — 95% CI [93.2%, 96.2%].

We note an increase in coverage rate as compared to the linear programming based optimizer using personalized antibiograms (85.9% — 95% CI [83.6 %, 88.3%]) and the actual clinician allocation (84.3% — 95% CI [81.8 %, 86.9%]) which makes sense given that in the above two conditions strictly more information is available to leverage in making the antibiotic selection. Neither of these conditions fairly replicate the real life empiric decision condition where neither the organism identity nor the gram stain is known, however they are useful experimental conditions to simulate to demonstrate the utility of the linear programming based optimization framework when paired with potential future rapid diagnostics technology that would allow knowledge of gram stain and or species identity at empiric antibiotic selection time.

## Supplementary Note 4

In this section we describe and show results for an additional subgroup analysis conducted to compare the performance of our personalized antibiogram against the performance of an algorithm intended to mimic clinical practice guidelines. We performed this analysis on the subgroup of patients in our Stanford cohort who had positive urine cultures and no other positive microbial cultures (ie blood, fluid). This reduced the size of our test set from $N_{test}$=770 to $N_{UTI}$=476. We simulated an antibiotic allocation to this subset of our test set using a rules based system intended to mimic our local clinical practice guidelines for treating patients hospitalized with urinary tract infection. The rules based algorithm was defined as follows.

- Order ceftriaxone if absolute neutrophil count greater than or equal to 1,000 neutrophils / $\mu$L and patient had no history of ceftriaxone resistance reported in their EHR.
- Order piperacillin/tazobactam if the patient had a history of ceftriaxone resistance and no prior resistance to piperacillin/tazobactam.
- Order piperacillin/tazobactam if the patient had an absolute neutrophil count less than 1,000 neutrophils / $\mu$L and no prior resistance to piperacillin/tazobactam.
- Otherwise order meropenem.

After applying these rules to the set of 476 patients in our test set hospitalized with urinary tract infection the distribution of antibiotic allocations was as follows. Ceftriaxone was allocated by the guideline based algorithm 211 times. Piperacillin/tazobactam was allocated 247 times, and meropenem was allocated 18 times. The resulting coverage rate in the subgroup was 84.7% — 95% CI [81.3%, 87.6%].

To create a fair comparison, we benchmarked this algorithm against the personalized antibiogram based prescribing procedure by restricting the budget parameters of the linear programming formulation to match the antibiotic distribution used by the guideline based algorithm. Specifically, we forced the optimizer to similarly prescribe ceftriaxone 211 times, piperacillin/tazobactam 247 times and meropenem

18 times. With these budget parameters, the personalized antibiogram approach achieved a coverage rate of 89.1% — 95% CI [86.3%, 91.6%] on this subgroup of patients.

We further compared these two coverage rates to the coverage rate achieved by clinicians. We note however that this comparison isn't apples-to-apples because clinicians used a more diverse set of antibiotics, with a larger amount of ceftriaxone. The coverage rate achieved by clinicians on this subgroup of patients was 81.5% — 95% CI [78.2%, 84.9%]. The distribution of antibiotics used by clinicians was as follows: ceftriaxone=335, vancomycin + piperacillin-tazobactam=51, piperacillin-tazobactam=36, vancomycin + ceftriaxone=10, cefepime=9, vancomycin+cefepime=8, ciprofloxacin=7, cefazolin=7, vancomycin + meropenem=5, meropenem=5, vancomycin=3. We note that the more diverse set of antibiotics likely is due to the fact that although the particular syndrome was clear after the fact, at the time of empiric antibiotic selection other syndromes and pathogens were potentially suspected.

This finding demonstrates promising utility of our personalized antibiogram based optimization procedure compared to a guideline based approach — especially in a setting when syndromes and pathogens (to which guidelines are tailored) are only suspected and not known.

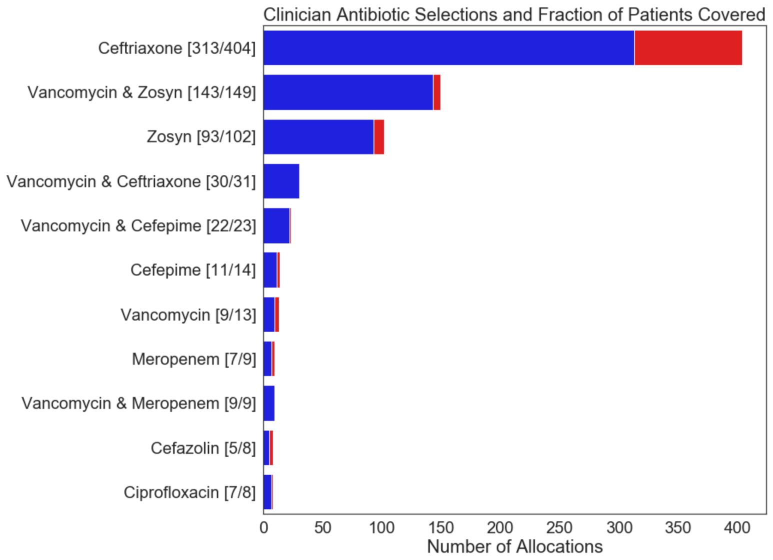


Supplementary Figure 2: The number of times particular antibiotic selections were administered to patients in our one year test set (2019), along with the fraction of patients who were covered by the chosen treatment (blue) and those who were not (red).

Supplementary Figure 3: In the following four pages, we show results of simulations where the budgets of broader spectrum antibiotics were incrementally decreased in favor of larger budgets of narrower spectrum antibiotics across all (broader, narrower) spectrum pairs in the Stanford dataset.

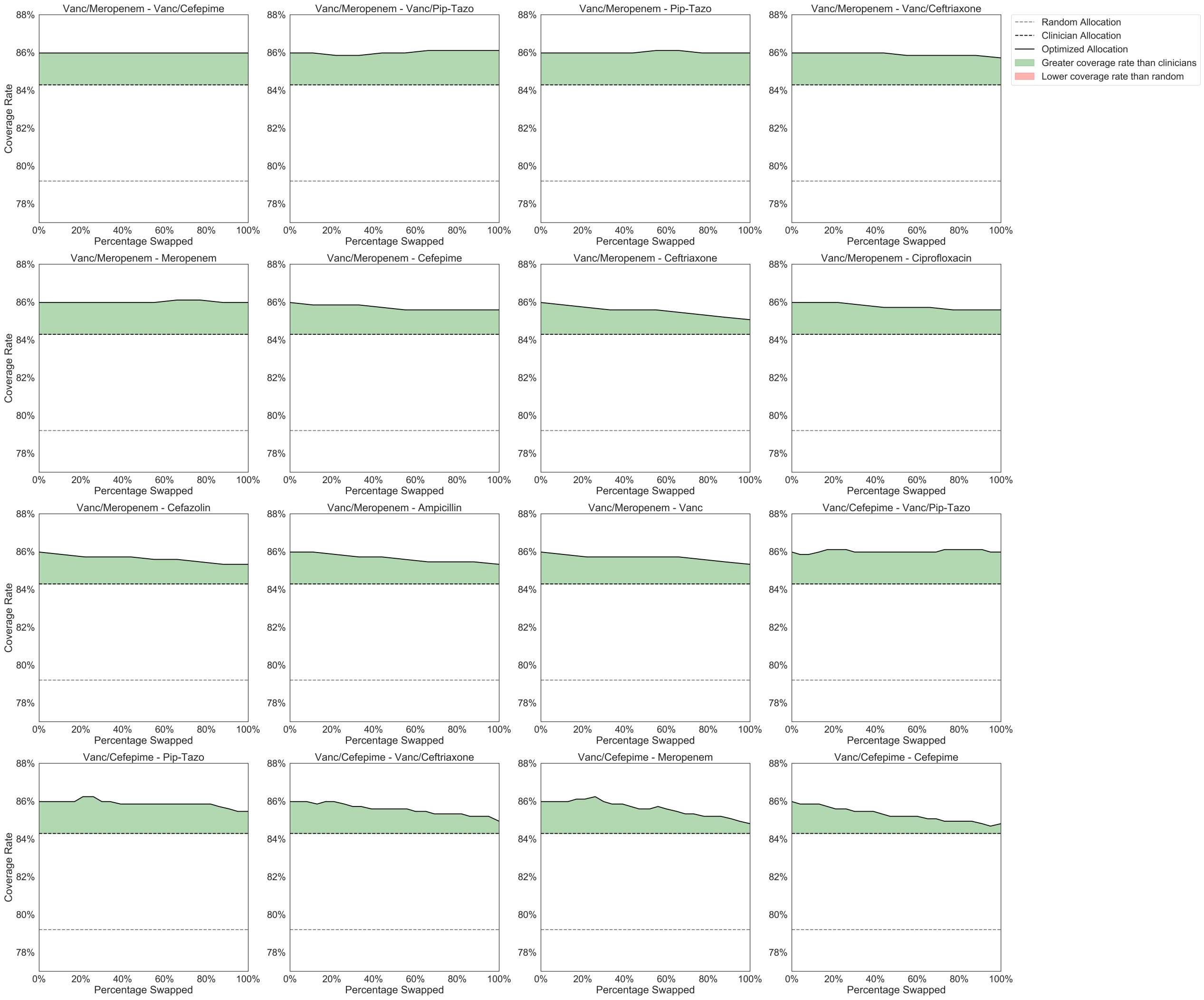

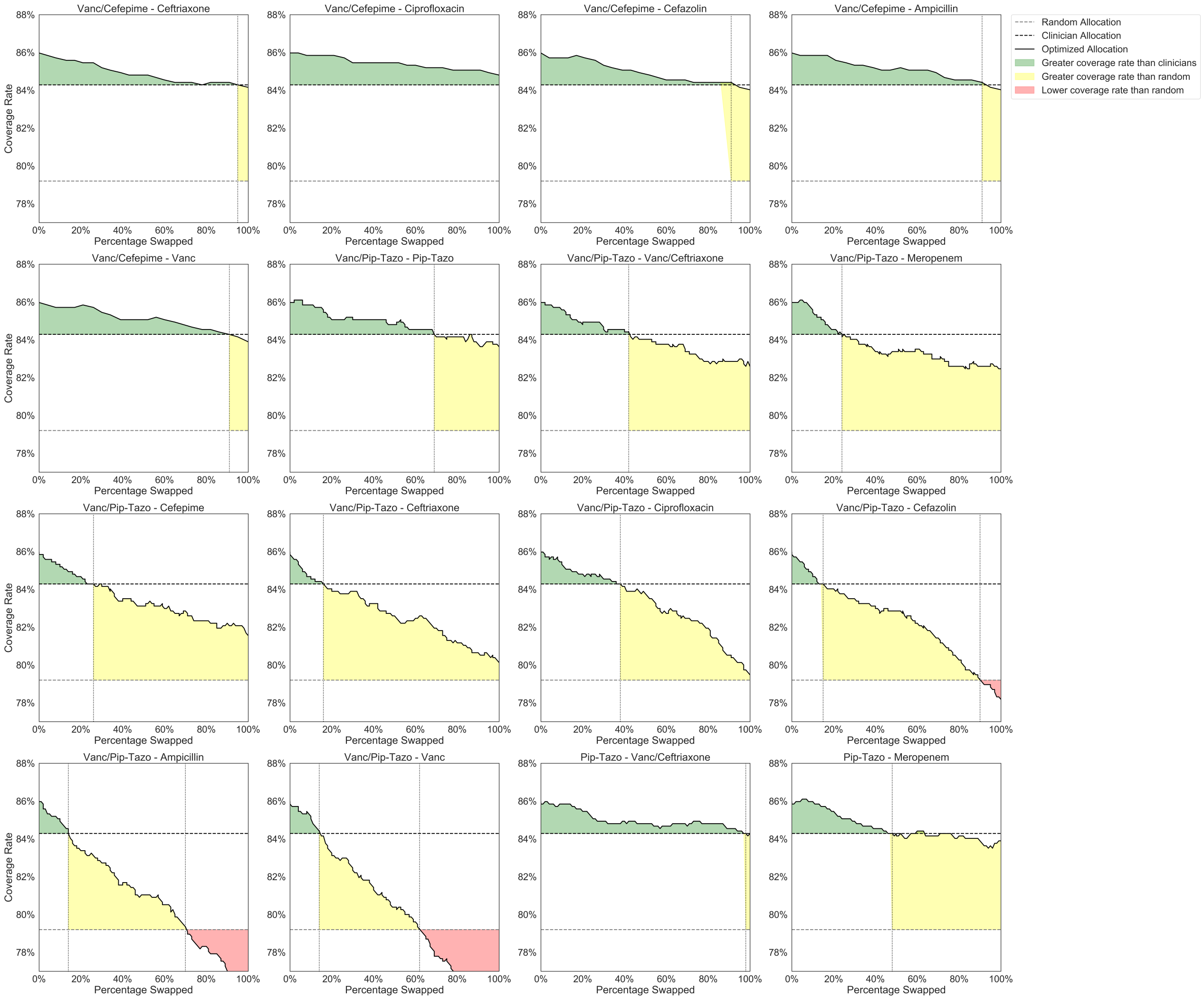

Vanc/Cefepime - Ceftriaxone
Vanc/Cefepime - Ciprofloxacin
Vanc/Cefepime - Cefazolin
Vanc/Cefepime - Ampicillin
Vanc/Cefepime - Vanc
Vanc/Pip-Tazo - Pip-Tazo
Vanc/Pip-Tazo - Vanc/Ceftriaxone
Vanc/Pip-Tazo - Meropenem
Vanc/Pip-Tazo - Cefepime
Vanc/Pip-Tazo - Ceftriaxone
Vanc/Pip-Tazo - Ciprofloxacin
Vanc/Pip-Tazo - Cefazolin
Vanc/Pip-Tazo - Ampicillin
Vanc/Pip-Tazo - Vanc
Pip-Tazo - Vanc/Ceftriaxone
Pip-Tazo - Meropenem
Coverage Rate
Percentage Swapped
Random Allocation
Clinician Allocation
Optimized Allocation
Greater coverage rate than clinicians
Greater coverage rate than random
Lower coverage rate than random

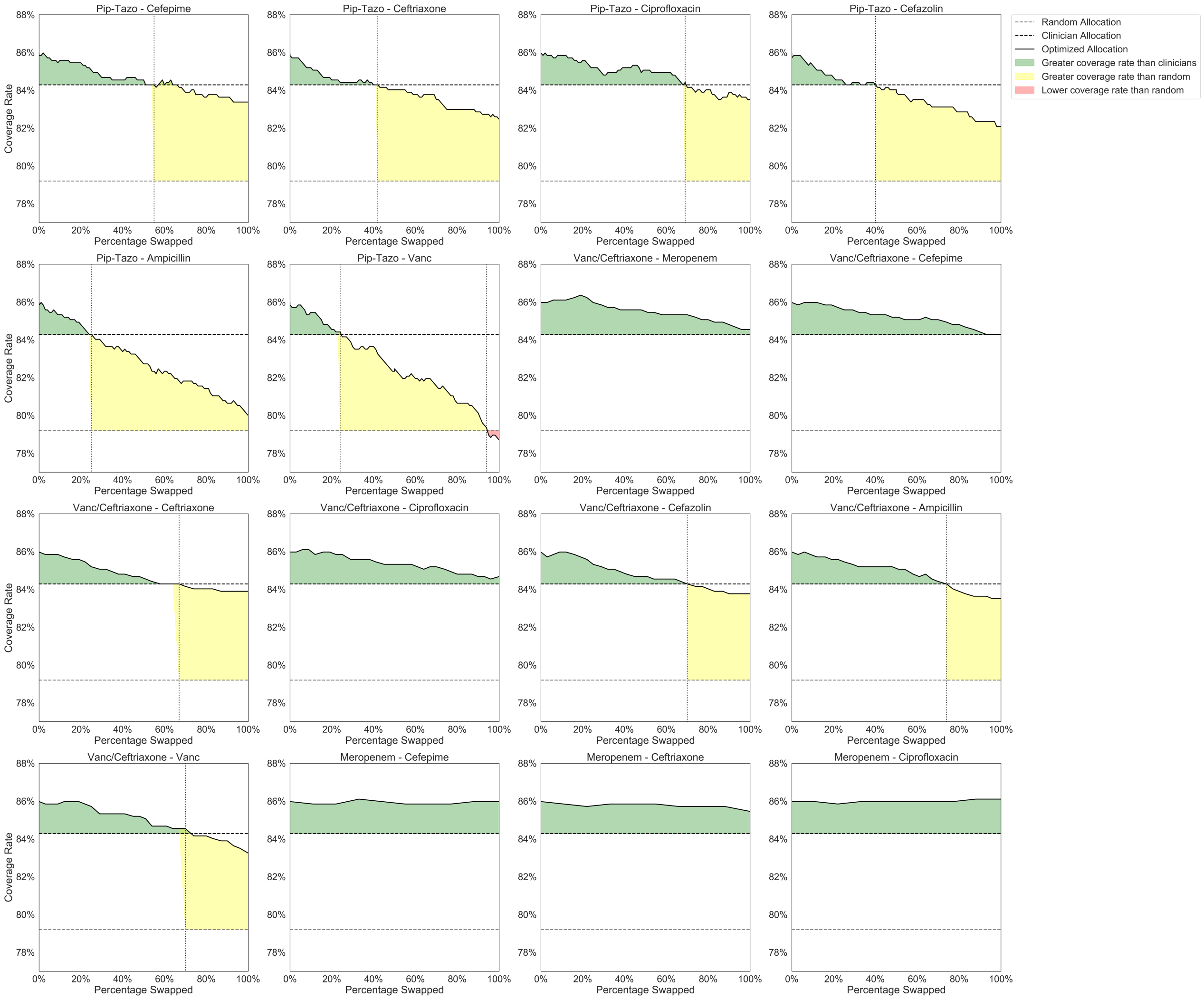
Pip-Tazo - Cefepime
Pip-Tazo - Ceftriaxone
Pip-Tazo - Ciprofloxacin
Pip-Tazo - Cefazolin
Pip-Tazo - Ampicillin
Pip-Tazo - Vanc
Vanc/Ceftriaxone - Meropenem
Vanc/Ceftriaxone - Cefepime
Vanc/Ceftriaxone - Ceftriaxone
Vanc/Ceftriaxone - Ciprofloxacin
Vanc/Ceftriaxone - Cefazolin
Vanc/Ceftriaxone - Ampicillin
Vanc/Ceftriaxone - Vanc
Meropenem - Cefepime
Meropenem - Ceftriaxone
Meropenem - Ciprofloxacin
Coverage Rate
Percentage Swapped
88%
86%
84%
82%
80%
78%
0%
20%
40%
60%
80%
100%
Random Allocation
Clinician Allocation
Optimized Allocation
Greater coverage rate than clinicians
Greater coverage rate than random
Lower coverage rate than random

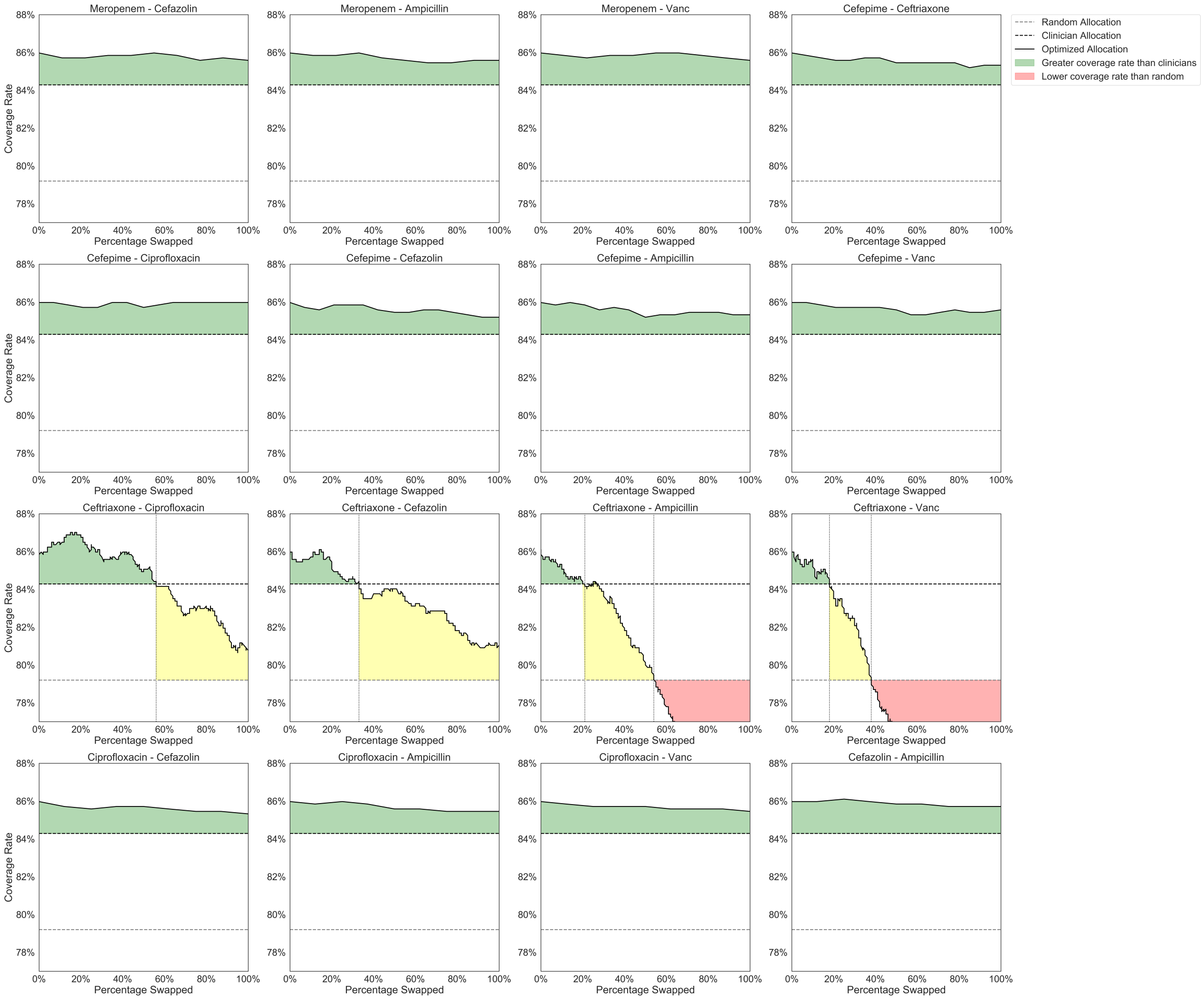
Meropenem - Cefazolin
Meropenem - Ampicillin
Meropenem - Vanc
Cefepime - Ceftriaxone
Cefepime - Ciprofloxacin
Cefepime - Cefazolin
Cefepime - Ampicillin
Cefepime - Vanc
Ceftriaxone - Ciprofloxacin
Ceftriaxone - Cefazolin
Ceftriaxone - Ampicillin
Ceftriaxone - Vanc
Ciprofloxacin - Cefazolin
Ciprofloxacin - Ampicillin
Ciprofloxacin - Vanc
Cefazolin - Ampicillin
Random Allocation
Clinician Allocation
Optimized Allocation
Greater coverage rate than clinicians
Lower coverage rate than random
Coverage Rate
Percentage Swapped
88%
86%
84%
82%
80%
78%
0%
20%
40%
60%
80%
100%

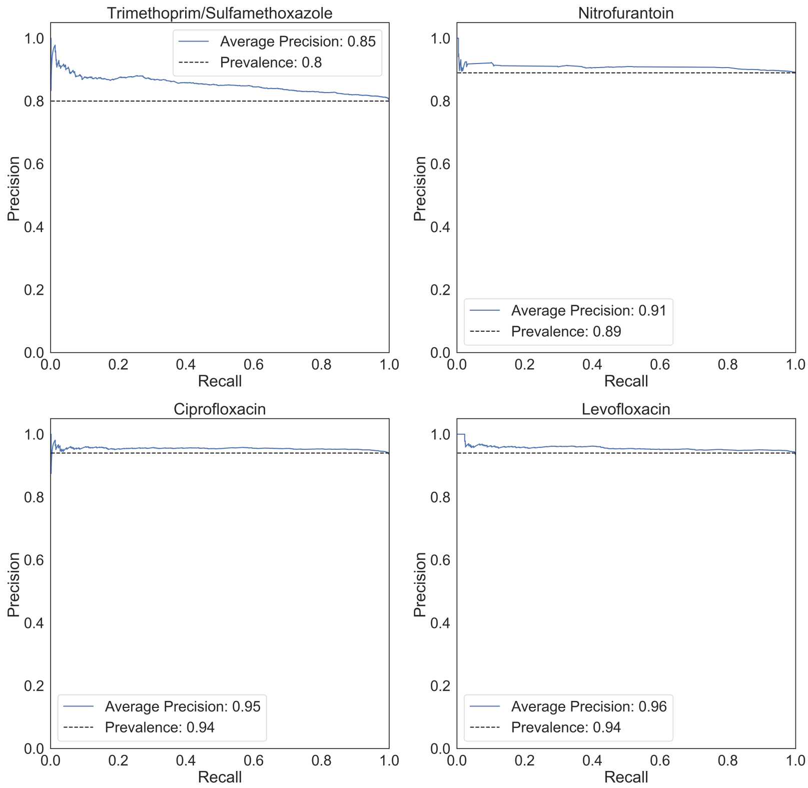


Supplementary Figure 4: Precision recall curves for each of the twelve personalized antibiogram models trained using the Boston Dataset.

Supplementary Table 1: Performance of classifiers on validation set with best hyperparameters

| **Antibiotic Selection** | **Model Class** | **AUROC** | **Best Hyperparameters** |
|---|---|---|---|
| Vancomycin | lasso | 0.69 [0.65, 0.73] | {'C': 0.1} |
| | ridge | 0.72 [0.69, 0.76] | {'C': 0.001} |
| | random_forest | 0.73 [0.69, 0.76] | {'max_features': 'sqrt', 'min_samples_split': 2} |
| | lightgbm | 0.74 [0.70, 0.77] | {'learning rate': 0.01, 'num leaves': 64, 'boosting rounds': 222} |
| Ampicillin | lasso | 0.60 [0.57, 0.63] | {'C': 0.01} |
| | ridge | 0.60 [0.57, 0.63] | {'C': 0.001} |
| | random_forest | 0.61 [0.58, 0.64] | {'max_features': 'sqrt', 'min_samples_split': 2} |
| | lightgbm | 0.61 [0.58, 0.64] | {'learning_rate': 0.05, 'num_leaves': 64, 'boosting_rounds': 33} |
| Cefazolin | lasso | 0.60 [0.57, 0.63] | {'C': 0.1} |
| | ridge | 0.62 [0.59, 0.65] | {'C': 0.0001} |
| | random_forest | 0.64 [0.61, 0.67] | {'max_features': 'sqrt', 'min_samples_split': 2} |
| | lightgbm | 0.65 [0.62, 0.69] | {'learning_rate': 0.01, 'num_leaves': 64, 'boosting_rounds': 167} |
| Ceftriaxone | lasso | 0.62 [0.58, 0.65] | {'C': 0.1} |
| | ridge | 0.65 [0.61, 0.69] | {'C': 0.001} |
| | random_forest | 0.68 [0.64, 0.71] | {'max_features': 'sqrt', 'min_samples_split': 2} |
| | lightgbm | 0.68 [0.65, 0.72] | {'learning_rate': 0.01, 'num_leaves': 64, 'boosting_rounds': 220} |
| Cefepime | lasso | 0.57 [0.53, 0.61] | {'C': 0.1} |
| | ridge | 0.61 [0.57, 0.65] | {'C': 0.0001} |
| | random_forest | 0.66 [0.62, 0.70] | {'max_features': 'sqrt', 'min_samples_split': 2} |
| | lightgbm | 0.63 [0.59, 0.67] | {'learning_rate': 0.05, 'num_leaves': 64, 'boosting_rounds': 45} |
| Pip-Tazo | lasso | 0.59 [0.53, 0.64] | {'C': 0.1} |
| | ridge | 0.56 [0.51, 0.61] | {'C': 1e-05} |
| | random_forest | 0.63 [0.57, 0.67] | {'max_features': 'log2', 'min_samples_split': 2} |
| | lightgbm | 0.60 [0.55, 0.64] | {'learning_rate': 0.01, 'num_leaves': 64 'boosting_rounds': 125} |
| Ciprofloxacin | lasso | 0.60 [0.57, 0.63] | {'C': 0.1} |
| | ridge | 0.60 [0.56, 0.63] | {'C': 0.001} |
| | random_forest | 0.61 [0.58, 0.65] | {'max_features': 'sqrt', 'min_samples_split': 2} |
| | lightgbm | 0.59 [0.55, 0.62] | {'learning_rate': 0.01, 'num_leaves': 64 'boosting_rounds': 97} |
| Meropenem | lasso | 0.57 [0.53, 0.62] | {'C': 0.1} |
| | ridge | 0.61 [0.57, 0.65] | {'C': 0.0001} |
| | random_forest | 0.66 [0.62, 0.70] | {'max_features': 'sqrt', 'min_samples_split': 2} |
| | lightgbm | 0.65 [0.61, 0.69] | {'learning_rate': 0.01, 'num_leaves': 64, 'boosting_rounds': 184} |
| Vancomycin & Meropenem | lasso | 0.51 [0.40, 0.61] | {'C': 1.0} |
| | ridge | 0.58 [0.47, 0.68] | {'C': 0.0001} |
| | random_forest | 0.66 [0.54, 0.78] | {'max_features': 'log2', 'min_samples_split': 2} |
| | lightgbm | 0.68 [0.57, 0.78] | {'learning_rate': 0.01, 'num_leaves': 64, 'boosting_rounds': 61} |
| Vancomycin & Pip-Tazo | lasso | 0.54 [0.47, 0.62] | {'C': 0.1} |
| | ridge | 0.56 [0.49, 0.63] | {'C': 1e-05} |
| | random_forest | 0.61 [0.55, 0.67] | {'max_features': 'log2', 'min_samples_split': 50} |
| | lightgbm | 0.53 [0.45, 0.60] | {'learning_rate': 0.1, 'num_leaves': 64, 'boosting_rounds': 5} |
| Vancomycin & Cefepime | lasso | 0.48 [0.40, 0.57] | {'C': 1.0} |
| | ridge | 0.66 [0.58, 0.74] | {'C': 0.001} |
| | random_forest | 0.68 [0.60, 0.77] | {'max_features': 'log2', 'min_samples_split': 2} |
| | lightgbm | 0.67 [0.58, 0.76] | {'learning_rate': 0.01, 'num_leaves': 64, 'boosting_rounds': 54} |
| Vancomycin & Ceftriaxone | lasso | 0.59 [0.54, 0.63] | {'C': 0.1} |
| | ridge | 0.62 [0.58, 0.65] | {'C': 0.0001} |
| | random_forest | 0.68 [0.64, 0.72] | {'max_features': 'log2', 'min_samples_split': 50} |
| | lightgbm | 0.70 [0.66, 0.74] | {'learning_rate': 0.01, 'num_leaves': 64, 'boosting_rounds': 150} |

Supplementary Table 2: Number of distinct features by feature category used by the Stanford personalized antibiogram models.

| **Feature Category** | **Number of Distinct Features** |
|---|---|
| Diagnosis Codes | 16696 |
| Medication Orders | 15965 |
| Microbial Culture Results | 3585 |
| Imaging Orders | 2580 |
| Lab Orders | 2421 |
| Procedure Orders | 956 |
| Lab Results | 538 |
| Flowsheets | 149 |
| Demographics | 125 |
| Microbiology Orders | 100 |
| Microbiology Culture Orders | 57 |
| Respiratory Care Orders | 46 |
| Department ID | 2 |
| **Total** | 43,220 |

Supplementary Table 3: Top five features ranked by feature importance for each of the twelve Stanford personalized antibiogram models.

| Antibiotic | Features | Feature Categories |
|---|---|---|
| Vancomycin | Age | Demographics |
| | Stanford ED | Department ID |
| | Urine Culture | Microbiology Culture Order |
| | Sedimentation Rate (ESR) | Lab Order |
| | Female | Demographics |
| Ampicillin | Age | Demographics |
| | Stanford ED | Department |
| | Escherichia coli Ampicillin Resistant | Microbial Culture Results |
| | Z87.440: Personal History of Urinary Tract Infection | Diagnosis Codes |
| | White | Demographics |
| Cefazolin | Age | Demographics |
| | Stanford ED | Department ID |
| | Female | Demographics |
| | Escherichia coli Cefazolin Resistant | Microbial Culture Results |
| | Urine Culture | Microbiology Culture Order |
| Ceftriaxone | Age | Demographics |
| | Stanford ED | Department ID |
| | Female | Demographics |
| | Escherichia coli Cefazolin Resistant | Microbial Culture Results |
| | Pseudomonas aeruginosa Cefepime Susceptible | Microbial Culture Results |
| Cefepime | Age | Demographics |
| | Stanford ED | Department ID |
| | Male | Demographics |
| | Female | Demographics |
| | Medicare | Demographics |
| Pip-Tazo | Age | Demographics |
| | Stanford ED | Department ID |
| | Female | Demographics |
| | BUN 9th Decile | Lab Results |
| | CL 4th Decile | Lab Results |
| Ciprofloxacin | Escherichia coli Levofloxacin Resistant | Microbial Culture Results |
| | Escherichia coli Ciprofloxacin Resistant | Microbial Culture Results |
| | Stanford ED | Department ID |
| | Age | Demographics |
| | Valley Care ED | Department ID |
| Meropenem | Stanford ED | Department ID |
| | Age | Demographics |
| | Female | Demographics |
| | Anaerobic Culture | Microbiology Culture Order |
| | Bisacodyl 10 MG PR SUPP | Medication Order |
| Vancomycin & Meropenem | Stanford ED | Department ID |
| | Age | Demographics |
| | Anaerobic Culture | Microbiology Culture Order |
| | PHV 8th Decile | Lab Results |
| | Acetaminophen 325 MG PO TABS | Medication Order |
| Vancomycin & Pip-Tazo | Stanford ED | Department |
| | Age | Demographics |
| | R26.9: Unspecified Abnormalities of Gait | Diagnosis Codes |
| | CL 1st Decile | Lab Results |
| | TBIL 2nd Decile | Lab Results |
| Vancomycin & Cefepime | Stanford ED | Department ID |
| | Escherichia coli Imipenem Susceptible | Microbial Culture Results |
| | Malaria Peripheral Smear And Antigen Screen | Lab |
| | R10.84: Generalized Abdominal Pain | Diagnosis Codes |
| | Constulose 10 GRAM/15 ML PO SOLN | Medication Orders |
| Vancomycin & Ceftriaxone | Age | Demographics |
| | Stanford ED | Department ID |
| | Escherichia coli Ceftriaxone Resistant | Microbial Culture Results |
| | Escherichia coli Gentamicin Susceptible | Microbial Culture Results |
| | TCO2A 10th Decile | Lab Results |

Supplementary Table 4: Classifier performance on test set by whether patient had observations seen during model training

| **Antibiotic Selection** | **AUROC** | | |
|---|---|---|---|
| | **All Observations N=1320** | **Patients Seen In Training N=216** | **Patients Not Seen In Training N=1104** |
| Vancomycin | 0.72 [0.68, 0.75] | 0.64 [0.53, 0.74] | 0.72 [0.69, 0.76] |
| Ampicillin | 0.62 [0.59, 0.65] | 0.65 [0.57, 0.72] | 0.61 [0.57, 0.64] |
| Cefazolin | 0.67 [0.64, 0.70] | 0.72 [0.66, 0.78] | 0.66 [0.63, 0.70] |
| Ceftriaxone | 0.69 [0.66, 0.72] | 0.75 [0.68, 0.82] | 0.67 [0.64, 0.71] |
| Cefepime | 0.65 [0.61, 0.69] | 0.70 [0.61, 0.78] | 0.64 [0.60, 0.67] |
| Pip-Tazo | 0.64 [0.59, 0.69] | 0.74 [0.62, 0.84] | 0.62 [0.56, 0.68] |
| Ciprofloxacin | 0.61 [0.58, 0.64] | 0.67 [0.60, 0.74] | 0.60 [0.57, 0.63] |
| Meropenem | 0.69 [0.65, 0.72] | 0.69 [0.61, 0.78] | 0.68 [0.64, 0.72] |
| Vancomycin & Meropenem | 0.73 [0.65, 0.80] | 0.77 [0.59, 0.94] | 0.73 [0.63, 0.81] |
| Vancomycin & Pip-Tazo | 0.70 [0.62, 0.77] | 0.74 [0.56, 0.89] | 0.68 [0.59, 0.77] |
| Vancomycin & Cefepime | 0.70 [0.62, 0.77] | 0.82 [0.65, 0.95] | 0.69 [0.60, 0.76] |
| Vancomycin & Ceftriaxone | 0.67 [0.63, 0.71] | 0.70 [0.60, 0.78] | 0.66 [0.61, 0.70] |

Supplementary Table 5: Vancomycin classifier performance stratified by demographics groups. When only one class exists in a given strata, the AUROC is listed as NaN.

| **Stratified By** | **Group** | **# Positive Examples** | **# Total Examples** | **AUROC** |
|---|---|---|---|---|
| | **All Observations** | **302** | **1320** | **0.72 [0.68, 0.75]** |
| Age | (18, 30) | 16 | 49 | 0.67 [0.51, 0.82] |
| | (31, 50) | 40 | 132 | 0.72 [0.62, 0.82] |
| | (51, 60) | 35 | 133 | 0.67 [0.56, 0.76] |
| | (61, 70) | 53 | 232 | 0.74 [0.65, 0.82] |
| | (71, 80) | 77 | 308 | 0.71 [0.64, 0.78] |
| | (81, 90) | 81 | 466 | 0.70 [0.63, 0.76] |
| Race | Other | 51 | 251 | 0.68 [0.59, 0.76] |
| | White | 184 | 757 | 0.71 [0.66, 0.75] |
| | Asian | 33 | 201 | 0.73 [0.64, 0.82] |
| | Black | 24 | 69 | 0.80 [0.69, 0.90] |
| | Pacific Islander | 8 | 30 | 0.72 [0.50, 0.89] |
| | Unknown | 0 | 7 | NaN |
| | Native American | 2 | 5 | 1.00 [1.00, 1.00] |
| Department ID | Valley Care ED | 86 | 465 | 0.75 [0.68, 0.80] |
| | Stanford ED | 216 | 855 | 0.70 [0.66, 0.74] |
| Sex | Female | 134 | 793 | 0.68 [0.62, 0.73] |
| | Male | 168 | 527 | 0.71 [0.66, 0.76] |
| Ethnicity | Non-Hispanic | 259 | 1117 | 0.73 [0.69, 0.76] |
| | Hispanic/Latino | 41 | 195 | 0.66 [0.56, 0.76] |
| | Unknown | 2 | 8 | 0.59 [0.00, 1.00] |
| Language | English | 267 | 1112 | 0.72 [0.68, 0.76] |
| | Non-English | 35 | 208 | 0.68 [0.57, 0.78] |
| Insurance Payer | Other | 165 | 615 | 0.71 [0.66, 0.76] |
| | Medicare | 124 | 651 | 0.71 [0.66, 0.76] |
| | Medi-Cal | 13 | 54 | 0.80 [0.64, 0.93] |

Supplementary Table 6: Ampicillin classifier performance stratified by demographics groups

| Stratified By | Group | # Positive Examples | # Total Examples | AUROC |
|---|---|---|---|---|
| | **All Observations** | **564** | **1320** | **0.62 [0.59, 0.65]** |
| Age | (18, 30) | 24 | 49 | 0.65 [0.48, 0.79] |
| | (31, 50) | 66 | 132 | 0.63 [0.53, 0.73] |
| | (51, 60) | 57 | 133 | 0.55 [0.46, 0.65] |
| | (61, 70) | 88 | 232 | 0.71 [0.64, 0.78] |
| | (71, 80) | 131 | 308 | 0.63 [0.57, 0.69] |
| | (81, 90) | 198 | 466 | 0.56 [0.51, 0.61] |
| Race | Other | 104 | 251 | 0.61 [0.54, 0.67] |
| | White | 330 | 757 | 0.62 [0.58, 0.66] |
| | Asian | 73 | 201 | 0.60 [0.52, 0.68] |
| | Black | 35 | 69 | 0.72 [0.60, 0.84] |
| | Pacific Islander | 16 | 30 | 0.66 [0.45, 0.85] |
| | Unknown | 3 | 7 | 0.51 [0.00, 1.00] |
| | Native American | 3 | 5 | 0.68 [0.00, 1.00] |
| Department ID | Valley Care ED | 172 | 465 | 0.57 [0.52, 0.63] |
| | Stanford ED | 392 | 855 | 0.64 [0.60, 0.67] |
| Sex | Female | 343 | 793 | 0.61 [0.57, 0.65] |
| | Male | 221 | 527 | 0.64 [0.59, 0.69] |
| Ethnicity | Non-Hispanic | 482 | 1117 | 0.62 [0.59, 0.66] |
| | Hispanic/Latino | 79 | 195 | 0.62 [0.53, 0.70] |
| | Unknown | 3 | 8 | 0.54 [0.07, 1.00] |
| Language | English | 487 | 1112 | 0.63 [0.60, 0.66] |
| | Non-English | 77 | 208 | 0.56 [0.47, 0.65] |
| Insurance Payer | Other | 274 | 615 | 0.66 [0.62, 0.70] |
| | Medicare | 265 | 651 | 0.58 [0.53, 0.62] |
| | Medi-Cal | 25 | 54 | 0.65 [0.50, 0.78] |

Supplementary Table 7: Cefazolin classifier performance stratified by demographics groups

| Stratified By | Group | # Positive Examples | # Total Examples | AUROC |
|---|---|---|---|---|
| | **All Observations** | **780** | **1320** | **0.67 [0.65, 0.70]** |
| Age | (18, 30) | 29 | 49 | 0.65 [0.49, 0.80] |
| | (31, 50) | 90 | 132 | 0.71 [0.62, 0.80] |
| | (51, 60) | 82 | 133 | 0.65 [0.54, 0.75] |
| | (61, 70) | 122 | 232 | 0.74 [0.68, 0.80] |
| | (71, 80) | 168 | 308 | 0.66 [0.60, 0.72] |
| | (81, 90) | 289 | 466 | 0.64 [0.59, 0.69] |
| Race | Other | 143 | 251 | 0.72 [0.65, 0.77] |
| | White | 442 | 757 | 0.66 [0.62, 0.70] |
| | Asian | 123 | 201 | 0.68 [0.60, 0.75] |
| | Black | 42 | 69 | 0.63 [0.48, 0.77] |
| | Pacific Islander | 20 | 30 | 0.88 [0.72, 0.98] |
| | Unknown | 7 | 7 | NaN |
| | Native American | 3 | 5 | 0.65 [0.00, 1.00] |
| Department ID | Valley Care ED | 282 | 465 | 0.68 [0.63, 0.74] |
| | Stanford ED | 498 | 855 | 0.67 [0.63, 0.71] |
| Sex | Female | 491 | 793 | 0.68 [0.64, 0.72] |
| | Male | 289 | 527 | 0.65 [0.60, 0.69] |
| Ethnicity | Non-Hispanic | 662 | 1117 | 0.67 [0.64, 0.70] |
| | Hispanic/Latino | 113 | 195 | 0.71 [0.63, 0.78] |
| | Unknown | 5 | 8 | 1.00 [1.00, 1.00] |
| Language | English | 665 | 1112 | 0.66 [0.63, 0.70] |
| | Non-English | 115 | 208 | 0.72 [0.65, 0.79] |
| Insurance Payer | Other | 377 | 615 | 0.69 [0.64, 0.73] |
| | Medicare | 370 | 651 | 0.66 [0.62, 0.71] |
| | Medi-Cal | 33 | 54 | 0.67 [0.52, 0.82] |

Supplementary Table 8: Ciprofloxacin classifier performance stratified by demographics groups

| **Stratified By** | **Group** | **# Positive Examples** | **# Total Examples** | **AUROC** |
|---|---|---|---|---|
| | **All Observations** | **828** | **1320** | **0.61 [0.58, 0.64]** |
| Age | (18, 30) | 21 | 49 | 0.72 [0.56, 0.85] |
| | (31, 50) | 91 | 132 | 0.61 [0.50, 0.71] |
| | (51, 60) | 78 | 133 | 0.61 [0.52, 0.71] |
| | (61, 70) | 144 | 232 | 0.59 [0.51, 0.66] |
| | (71, 80) | 182 | 308 | 0.62 [0.56, 0.68] |
| | (81, 90) | 312 | 466 | 0.60 [0.54, 0.65] |
| Race | Other | 149 | 251 | 0.62 [0.55, 0.69] |
| | White | 484 | 757 | 0.62 [0.58, 0.66] |
| | Asian | 128 | 201 | 0.60 [0.51, 0.68] |
| | Black | 39 | 69 | 0.56 [0.42, 0.70] |
| | Pacific Islander | 20 | 30 | 0.47 [0.21, 0.75] |
| | Unknown | 5 | 7 | 0.70 [0.20, 1.00] |
| | Native American | 3 | 5 | 0.34 [0.00, 1.00] |
| Department ID | Valley Care ED | 275 | 465 | 0.61 [0.56, 0.66] |
| | Stanford ED | 553 | 855 | 0.61 [0.57, 0.65] |
| Sex | Female | 520 | 793 | 0.62 [0.58, 0.66] |
| | Male | 308 | 527 | 0.59 [0.54, 0.64] |
| Ethnicity | Non-Hispanic | 702 | 1117 | 0.61 [0.57, 0.64] |
| | Hispanic/Latino | 122 | 195 | 0.62 [0.54, 0.71] |
| | Unknown | 4 | 8 | 0.81 [0.44, 1.00] |
| Language | English | 699 | 1112 | 0.61 [0.58, 0.65] |
| | Non-English | 129 | 208 | 0.59 [0.50, 0.66] |
| Insurance Payer | Other | 388 | 615 | 0.60 [0.55, 0.64] |
| | Medicare | 412 | 651 | 0.62 [0.58, 0.67] |
| | Medi-Cal | 28 | 54 | 0.62 [0.48, 0.77] |

Supplementary Table 9: Ceftriaxone classifier performance stratified by demographics groups

| **Stratified By** | **Group** | **# Positive Examples** | **# Total Examples** | **AUROC** |
|---|---|---|---|---|
| | **All Observations** | **876** | **1320** | **0.69 [0.66, 0.72]** |
| Age | (18, 30) | 30 | 49 | 0.76 [0.61, 0.90] |
| | (31, 50) | 105 | 132 | 0.67 [0.56, 0.77] |
| | (51, 60) | 91 | 133 | 0.70 [0.60, 0.80] |
| | (61, 70) | 133 | 232 | 0.70 [0.63, 0.76] |
| | (71, 80) | 188 | 308 | 0.68 [0.62, 0.74] |
| | (81, 90) | 329 | 466 | 0.66 [0.61, 0.72] |
| Race | Other | 158 | 251 | 0.71 [0.64, 0.78] |
| | White | 500 | 757 | 0.67 [0.63, 0.71] |
| | Asian | 141 | 201 | 0.67 [0.59, 0.76] |
| | Black | 47 | 69 | 0.72 [0.58, 0.85] |
| | Pacific Islander | 20 | 30 | 0.80 [0.60, 0.96] |
| | Unknown | 7 | 7 | NaN |
| | Native American | 3 | 5 | 0.33 [0.00, 1.00] |
| Department ID | Valley Care ED | 306 | 465 | 0.70 [0.65, 0.75] |
| | Stanford ED | 570 | 855 | 0.68 [0.65, 0.72] |
| Sex | Female | 557 | 793 | 0.69 [0.65, 0.73] |
| | Male | 319 | 527 | 0.66 [0.61, 0.71] |
| Ethnicity | Non-Hispanic | 744 | 1117 | 0.68 [0.65, 0.72] |
| | Hispanic/Latino | 127 | 195 | 0.71 [0.64, 0.79] |
| | Unknown | 5 | 8 | 1.00 [1.00, 1.00] |
| Language | English | 740 | 1112 | 0.69 [0.66, 0.72] |
| | Non-English | 136 | 208 | 0.67 [0.59, 0.75] |
| Insurance Payer | Other | 413 | 615 | 0.70 [0.65, 0.74] |
| | Medicare | 423 | 651 | 0.69 [0.64, 0.73] |
| | Medi-Cal | 40 | 54 | 0.69 [0.50, 0.84] |

Supplementary Table 10: Cefepime classifier performance stratified by demographics groups

| Stratified By | Group | # Positive Examples | # Total Examples | AUROC |
|---|---|---|---|---|
| | **All Observations** | **1055** | **1320** | **0.65 [0.61, 0.69]** |
| Age | (18, 30) | 36 | 49 | 0.75 [0.58, 0.90] |
| | (31, 50) | 117 | 132 | 0.80 [0.70, 0.89] |
| | (51, 60) | 113 | 133 | 0.54 [0.41, 0.67] |
| | (61, 70) | 173 | 232 | 0.66 [0.59, 0.73] |
| | (71, 80) | 233 | 308 | 0.61 [0.53, 0.68] |
| | (81, 90) | 383 | 466 | 0.66 [0.60, 0.73] |
| Race | Other | 206 | 251 | 0.62 [0.52, 0.71] |
| | White | 596 | 757 | 0.66 [0.61, 0.70] |
| | Asian | 164 | 201 | 0.67 [0.56, 0.76] |
| | Black | 55 | 69 | 0.72 [0.57, 0.85] |
| | Pacific Islander | 24 | 30 | 0.60 [0.33, 0.85] |
| | Unknown | 7 | 7 | NaN |
| | Native American | 3 | 5 | 0.49 [0.00, 1.00] |
| Department ID | Valley Care ED | 363 | 465 | 0.65 [0.59, 0.71] |
| | Stanford ED | 692 | 855 | 0.65 [0.60, 0.69] |
| Sex | Female | 657 | 793 | 0.67 [0.62, 0.72] |
| | Male | 398 | 527 | 0.60 [0.54, 0.66] |
| Ethnicity | Non-Hispanic | 888 | 1117 | 0.66 [0.62, 0.70] |
| | Hispanic/Latino | 162 | 195 | 0.56 [0.46, 0.67] |
| | Unknown | 5 | 8 | 0.93 [0.73, 1.00] |
| Language | English | 881 | 1112 | 0.65 [0.62, 0.69] |
| | Non-English | 174 | 208 | 0.62 [0.52, 0.72] |
| Insurance Payer | Other | 492 | 615 | 0.64 [0.59, 0.69] |
| | Medicare | 519 | 651 | 0.65 [0.60, 0.70] |
| | Medi-Cal | 44 | 54 | 0.69 [0.52, 0.84] |

Supplementary Table 11: Vancomycin & Ceftriaxone classifier performance stratified by demographics groups

| Stratified By | Group | # Positive Examples | # Total Examples | AUROC |
|---|---|---|---|---|
| | **All Observations** | **1064** | **1320** | **0.67 [0.63, 0.71]** |
| Age | (18, 30) | 36 | 49 | 0.70 [0.50, 0.88] |
| | (31, 50) | 113 | 132 | 0.79 [0.67, 0.88] |
| | (51, 60) | 106 | 133 | 0.66 [0.52, 0.80] |
| | (61, 70) | 171 | 232 | 0.64 [0.56, 0.72] |
| | (71, 80) | 244 | 308 | 0.64 [0.56, 0.72] |
| | (81, 90) | 394 | 466 | 0.67 [0.60, 0.74] |
| Race | Other | 186 | 251 | 0.68 [0.59, 0.77] |
| | White | 616 | 757 | 0.66 [0.61, 0.71] |
| | Asian | 164 | 201 | 0.61 [0.51, 0.72] |
| | Black | 61 | 69 | 0.78 [0.57, 0.96] |
| | Pacific Islander | 25 | 30 | 0.86 [0.71, 0.98] |
| | Unknown | 7 | 7 | NaN |
| | Native American | 5 | 5 | NaN |
| Department ID | Valley Care ED | 378 | 465 | 0.69 [0.61, 0.75] |
| | Stanford ED | 686 | 855 | 0.66 [0.61, 0.71] |
| Sex | Female | 655 | 793 | 0.68 [0.62, 0.73] |
| | Male | 409 | 527 | 0.65 [0.59, 0.71] |
| Ethnicity | Non-Hispanic | 907 | 1117 | 0.66 [0.62, 0.70] |
| | Hispanic/Latino | 151 | 195 | 0.69 [0.58, 0.79] |
| | Unknown | 6 | 8 | 0.92 [0.67, 1.00] |
| Language | English | 903 | 1112 | 0.66 [0.62, 0.71] |
| | Non-English | 161 | 208 | 0.69 [0.60, 0.78] |
| Insurance Payer | Other | 502 | 615 | 0.68 [0.62, 0.74] |
| | Medicare | 515 | 651 | 0.66 [0.60, 0.71] |
| | Medi-Cal | 47 | 54 | 0.60 [0.32, 0.85] |

Supplementary Table 12: Meropenem classifier performance stratified by demographics groups

| Stratified By | Group | # Positive Examples | # Total Examples | AUROC |
|---|---|---|---|---|
| | **All Observations** | **1083** | **1320** | **0.69 [0.65, 0.72]** |
| Age | (18, 30) | 39 | 49 | 0.73 [0.53, 0.90] |
| | (31, 50) | 121 | 132 | 0.74 [0.59, 0.89] |
| | (51, 60) | 113 | 133 | 0.54 [0.40, 0.67] |
| | (61, 70) | 181 | 232 | 0.73 [0.65, 0.80] |
| | (71, 80) | 239 | 308 | 0.69 [0.61, 0.76] |
| | (81, 90) | 390 | 466 | 0.68 [0.61, 0.74] |
| Race | Other | 210 | 251 | 0.64 [0.55, 0.73] |
| | White | 611 | 757 | 0.68 [0.63, 0.73] |
| | Asian | 173 | 201 | 0.77 [0.66, 0.87] |
| | Black | 55 | 69 | 0.68 [0.49, 0.83] |
| | Pacific Islander | 24 | 30 | 0.80 [0.62, 0.94] |
| | Unknown | 7 | 7 | NaN |
| | Native American | 3 | 5 | 0.34 [0.00, 1.00] |
| Department ID | Valley Care ED | 378 | 465 | 0.70 [0.64, 0.76] |
| | Stanford ED | 705 | 855 | 0.68 [0.63, 0.72] |
| Sex | Female | 671 | 793 | 0.71 [0.65, 0.76] |
| | Male | 412 | 527 | 0.63 [0.57, 0.68] |
| Ethnicity | Non-Hispanic | 912 | 1117 | 0.70 [0.66, 0.74] |
| | Hispanic/Latino | 165 | 195 | 0.59 [0.47, 0.70] |
| | Unknown | 6 | 8 | 0.83 [0.50, 1.00] |
| Language | English | 907 | 1112 | 0.70 [0.66, 0.74] |
| | Non-English | 176 | 208 | 0.61 [0.50, 0.71] |
| Insurance Payer | Other | 507 | 615 | 0.65 [0.59, 0.70] |
| | Medicare | 530 | 651 | 0.71 [0.65, 0.75] |
| | Medi-Cal | 46 | 54 | 0.84 [0.72, 0.95] |

Supplementary Table 13: Pip-Tazo classifier performance stratified by demographics groups

| Stratified By | Group | # Positive Examples | # Total Examples | AUROC |
|---|---|---|---|---|
| | **All Observations** | **1190** | **1320** | **0.64 [0.59, 0.70]** |
| Age | (18, 30) | 42 | 49 | 0.84 [0.69, 0.97] |
| | (31, 50) | 117 | 132 | 0.63 [0.46, 0.79] |
| | (51, 60) | 121 | 133 | 0.48 [0.30, 0.66] |
| | (61, 70) | 207 | 232 | 0.61 [0.48, 0.73] |
| | (71, 80) | 273 | 308 | 0.66 [0.57, 0.74] |
| | (81, 90) | 430 | 466 | 0.64 [0.54, 0.74] |
| Race | Other | 225 | 251 | 0.64 [0.51, 0.76] |
| | White | 677 | 757 | 0.64 [0.58, 0.71] |
| | Asian | 186 | 201 | 0.58 [0.40, 0.76] |
| | Black | 63 | 69 | 0.75 [0.49, 0.96] |
| | Pacific Islander | 27 | 30 | 0.52 [0.20, 0.81] |
| | Unknown | 7 | 7 | NaN |
| | Native American | 5 | 5 | NaN |
| Department ID | Valley Care ED | 406 | 465 | 0.66 [0.59, 0.74] |
| | Stanford ED | 784 | 855 | 0.61 [0.54, 0.68] |
| Sex | Female | 729 | 793 | 0.65 [0.58, 0.72] |
| | Male | 461 | 527 | 0.61 [0.53, 0.69] |
| Ethnicity | Non-Hispanic | 1004 | 1117 | 0.63 [0.58, 0.69] |
| | Hispanic/Latino | 179 | 195 | 0.69 [0.55, 0.82] |
| | Unknown | 7 | 8 | 1.00 [1.00, 1.00] |
| Language | English | 996 | 1112 | 0.63 [0.57, 0.69] |
| | Non-English | 194 | 208 | 0.71 [0.57, 0.85] |
| Insurance Payer | Other | 556 | 615 | 0.65 [0.58, 0.72] |
| | Medicare | 586 | 651 | 0.63 [0.56, 0.71] |
| | Medi-Cal | 48 | 54 | 0.58 [0.30, 0.83] |

Supplementary Table 14: Vancomycin & Pip-Tazo classifier performance stratified by demographics groups

| Stratified By | Group | # Positive Examples | # Total Examples | AUROC |
|---|---|---|---|---|
| | **All Observations** | **1267** | **1320** | **0.70 [0.62, 0.77]** |
| Age | (18, 30) | 47 | 49 | 0.78 [0.65, 0.89] |
| | (31, 50) | 124 | 132 | 0.76 [0.50, 0.95] |
| | (51, 60) | 130 | 133 | 0.92 [0.83, 0.99] |
| | (61, 70) | 221 | 232 | 0.68 [0.52, 0.83] |
| | (71, 80) | 297 | 308 | 0.76 [0.61, 0.88] |
| | (81, 90) | 448 | 466 | 0.60 [0.44, 0.74] |
| Race | Other | 239 | 251 | 0.63 [0.46, 0.81] |
| | White | 727 | 757 | 0.69 [0.58, 0.79] |
| | Asian | 193 | 201 | 0.75 [0.52, 0.91] |
| | Black | 67 | 69 | 0.94 [0.84, 1.00] |
| | Pacific Islander | 29 | 30 | 0.90 [0.76, 1.00] |
| | Unknown | 7 | 7 | NaN |
| | Native American | 5 | 5 | NaN |
| Department ID | Valley Care ED | 448 | 465 | 0.72 [0.58, 0.83] |
| | Stanford ED | 819 | 855 | 0.69 [0.58, 0.80] |
| Sex | Female | 762 | 793 | 0.66 [0.56, 0.77] |
| | Male | 505 | 527 | 0.75 [0.64, 0.85] |
| Ethnicity | Non-Hispanic | 1071 | 1117 | 0.68 [0.60, 0.77] |
| | Hispanic/Latino | 189 | 195 | 0.78 [0.64, 0.91] |
| | Unknown | 7 | 8 | 1.00 [1.00, 1.00] |
| Language | English | 1066 | 1112 | 0.68 [0.59, 0.77] |
| | Non-English | 201 | 208 | 0.80 [0.61, 0.93] |
| Insurance Payer | Other | 594 | 615 | 0.72 [0.60, 0.83] |
| | Medicare | 620 | 651 | 0.68 [0.57, 0.77] |
| | Medi-Cal | 53 | 54 | 0.92 [0.85, 0.98] |

Supplementary Table 15: Vancomycin & Cefepime classifier performance stratified by demographics groups

| Stratified By | Group | # Positive Examples | # Total Examples | AUROC |
|---|---|---|---|---|
| | **All Observations** | **1275** | **1320** | **0.70 [0.63, 0.78]** |
| Age | (18, 30) | 43 | 49 | 0.72 [0.49, 0.91] |
| | (31, 50) | 129 | 132 | 0.89 [0.73, 1.00] |
| | (51, 60) | 131 | 133 | 0.49 [0.27, 0.71] |
| | (61, 70) | 221 | 232 | 0.66 [0.47, 0.83] |
| | (71, 80) | 294 | 308 | 0.59 [0.45, 0.74] |
| | (81, 90) | 457 | 466 | 0.81 [0.69, 0.91] |
| Race | Other | 242 | 251 | 0.66 [0.46, 0.84] |
| | White | 731 | 757 | 0.74 [0.66, 0.82] |
| | Asian | 192 | 201 | 0.59 [0.38, 0.80] |
| | Black | 69 | 69 | NaN |
| | Pacific Islander | 29 | 30 | 0.93 [0.83, 1.00] |
| | Unknown | 7 | 7 | NaN |
| | Native American | 5 | 5 | NaN |
| Department ID | Valley Care ED | 444 | 465 | 0.69 [0.57, 0.80] |
| | Stanford ED | 831 | 855 | 0.74 [0.66, 0.82] |
| Sex | Female | 771 | 793 | 0.74 [0.64, 0.83] |
| | Male | 504 | 527 | 0.65 [0.51, 0.78] |
| Ethnicity | Non-Hispanic | 1078 | 1117 | 0.70 [0.63, 0.78] |
| | Hispanic/Latino | 191 | 195 | 0.59 [0.30, 0.92] |
| | Unknown | 6 | 8 | 1.00 [1.00, 1.00] |
| Language | English | 1072 | 1112 | 0.71 [0.63, 0.78] |
| | Non-English | 203 | 208 | 0.65 [0.37, 0.92] |
| Insurance Payer | Other | 591 | 615 | 0.68 [0.57, 0.78] |
| | Medicare | 631 | 651 | 0.70 [0.59, 0.80] |
| | Medi-Cal | 53 | 54 | 0.98 [0.94, 1.00] |

Supplementary Table 16: Vancomycin & Meropenem classifier performance stratified by demographics groups

| **Stratified By** | **Group** | **# Positive Examples** | **# Total Examples** | **AUROC** |
|---|---|---|---|---|
| | **All Observations** | **1287** | **1320** | **0.73 [0.65, 0.81]** |
| Age | (18, 30) | 45 | 49 | 0.71 [0.50, 0.89] |
| | (31, 50) | 129 | 132 | 0.93 [0.84, 1.00] |
| | (51, 60) | 129 | 133 | 0.63 [0.38, 0.82] |
| | (61, 70) | 226 | 232 | 0.69 [0.49, 0.87] |
| | (71, 80) | 297 | 308 | 0.73 [0.59, 0.85] |
| | (81, 90) | 461 | 466 | 0.70 [0.46, 0.93] |
| Race | Other | 243 | 251 | 0.63 [0.49, 0.74] |
| | White | 738 | 757 | 0.77 [0.67, 0.86] |
| | Asian | 197 | 201 | 0.65 [0.32, 0.99] |
| | Black | 68 | 69 | 0.81 [0.72, 0.90] |
| | Pacific Islander | 29 | 30 | 0.90 [0.79, 1.00] |
| | Unknown | 7 | 7 | NaN |
| | Native American | 5 | 5 | NaN |
| Department ID | Valley Care ED | 456 | 465 | 0.80 [0.65, 0.93] |
| | Stanford ED | 831 | 855 | 0.69 [0.59, 0.79] |
| Sex | Female | 776 | 793 | 0.72 [0.61, 0.83] |
| | Male | 511 | 527 | 0.74 [0.62, 0.84] |
| Ethnicity | Non-Hispanic | 1088 | 1117 | 0.73 [0.65, 0.82] |
| | Hispanic/Latino | 191 | 195 | 0.69 [0.60, 0.78] |
| | Unknown | 8 | 8 | NaN |
| Language | English | 1084 | 1112 | 0.75 [0.66, 0.83] |
| | Non-English | 203 | 208 | 0.63 [0.45, 0.80] |
| Insurance Payer | Other | 598 | 615 | 0.69 [0.57, 0.80] |
| | Medicare | 636 | 651 | 0.76 [0.64, 0.86] |
| | Medi-Cal | 53 | 54 | 0.96 [0.91, 1.00] |

Supplementary Table 17: Infections not covered by clinicians. GNRs = Gram Negative Rods, MRSA = *Methicillin Resistant Staph Aureus*

| **Culture Type** | **Infection Type** | **Number of Misses** |
|---|---|---|
| Urine Culture | Enterococcus species | 52 |
| Urine Culture | Lactose Fermenting GNRs | 39 |
| Urine Culture | Non Lactose Fermenting GNRs | 14 |
| Blood Culture | Lactose Fermenting GNRs | 8 |
| Blood Culture | Non Lactose Fermenting GNRs | 5 |
| Other Fluid Culture | Non Lactose Fermenting GNRs | 4 |
| Blood Culture | Enterococcus species | 3 |
| Blood Culture | Streptococcus species | 3 |
| Urine Culture | MRSA | 2 |
| Other Fluid Culture | Enterococcus species | 1 |
