# Supplementary material for: Personalized antibiograms for machine learning driven antibiotic selection
Source: Commun Med (Lond). 2022 Apr 8;2:38. doi: 10.1038/s43856-022-00094-8 (PMC9053259; doi:10.1038/s43856-022-00094-8)
Supplement: Supplementary file 5 — Description of Additional Supplementary Files [file 43856_2022_94_MOESM5_ESM.pdf]

Description of Additional Supplementary Files

File Name: Supplementary Data 1

Description: Source Data for Figure 4a and 4b

File Name: Supplementary Data 2

Description: Source Data for Figure 5a and 5b
