# Supplementary material for: Personalized antibiograms for machine learning driven antibiotic selection
Source: Commun Med (Lond). 2022 Apr 8;2:38. doi: 10.1038/s43856-022-00094-8 (PMC9053259; doi:10.1038/s43856-022-00094-8)
Supplement: Supplementary file 6 — Reporting Summary [file 43856_2022_94_MOESM6_ESM.pdf]

nature portfolio

Corresponding author(s): Conor K. Corbin

Last updated by author(s): Feb 3, 2022

Reporting Summary

Nature Portfolio wishes to improve the reproducibility of the work that we publish. This form provides structure for consistency and transparency in reporting. For further information on Nature Portfolio policies, see our Editorial Policies and the Editorial Policy Checklist.

Statistics

For all statistical analyses, confirm that the following items are present in the figure legend, table legend, main text, or Methods section.

n/a	Confirmed	
☐	☒	The exact sample size (n) for each experimental group/condition, given as a discrete number and unit of measurement
☐	☒	A statement on whether measurements were taken from distinct samples or whether the same sample was measured repeatedly
☐	☒	The statistical test(s) used AND whether they are one- or two-sided<br>*Only common tests should be described solely by name; describe more complex techniques in the Methods section.*
☐	☒	A description of all covariates tested
☐	☒	A description of any assumptions or corrections, such as tests of normality and adjustment for multiple comparisons
☐	☒	A full description of the statistical parameters including central tendency (e.g. means) or other basic estimates (e.g. regression coefficient) AND variation (e.g. standard deviation) or associated estimates of uncertainty (e.g. confidence intervals)
☐	☒	For null hypothesis testing, the test statistic (e.g. F, t, r) with confidence intervals, effect sizes, degrees of freedom and P value noted<br>*Give P values as exact values whenever suitable.*
☒	☐	For Bayesian analysis, information on the choice of priors and Markov chain Monte Carlo settings
☒	☐	For hierarchical and complex designs, identification of the appropriate level for tests and full reporting of outcomes
☒	☐	Estimates of effect sizes (e.g. Cohen's d, Pearson's r), indicating how they were calculated

Our web collection on statistics for biologists contains articles on many of the points above.

Software and code

Policy information about availability of computer code

Data collection	The Stanford data is made available through STARR, STAnford medicine Research data Repository [https://starr.stanford.edu/]. The data can be accessed for research purposes after Institutional Review Board approval via the Stanford Research Informatics Center. The Boston cohort has been made available through Physionet [https://physionet.org/content/antimicrobial-resistance-uti/1.0.0/] for credentialed users who sign the specified data usage agreement.
Data analysis	All custom code for this study has been made publicly available on GitHub: https://github.com/HealthRex/CDSS/tree/master/scripts/ER_Infection. A demo of the code has been made available and lists package requirements https://github.com/HealthRex/CDSS/blob/master/scripts/ER_Infection/notebooks/demo/Demo%20Personalized%20Antibiogram%20Linear%20Programming.ipynb. Installation instructions are made available https://github.com/HealthRex/CDSS

For manuscripts utilizing custom algorithms or software that are central to the research but not yet described in published literature, software must be made available to editors and reviewers. We strongly encourage code deposition in a community repository (e.g. GitHub). See the Nature Portfolio guidelines for submitting code & software for further information.

Data

Policy information about availability of data

All manuscripts must include a data availability statement. This statement should provide the following information, where applicable:

- Accession codes, unique identifiers, or web links for publicly available datasets
- A description of any restrictions on data availability
- For clinical datasets or third party data, please ensure that the statement adheres to our policy

The Stanford data is made available through STARR, STAnford medicine Research data Repository [https://starr.stanford.edu/]. The data can be accessed for

research purposes after Institutional Review Board approval via the Stanford Research Informatics Center. The Boston cohort has been made available through Physionet [https://physionet.org/content/antimicrobial-resistance-uti/1.0.0/] for credentialed users who sign the specified data usage agreement.

Field-specific reporting

Please select the one below that is the best fit for your research. If you are not sure, read the appropriate sections before making your selection.

☒ Life sciences ☐ Behavioural & social sciences ☐ Ecological, evolutionary & environmental sciences

For a reference copy of the document with all sections, see nature.com/documents/nr-reporting-summary-flat.pdf

Life sciences study design

All studies must disclose on these points even when the disclosure is negative.

Sample size	Stanford cohort had N=8,342 infections. Boston cohort had N=15,806 infections. Details of how cohorts were defined are listed in the methods section of the manuscript.
Data exclusions	See method section titled "Cohort descriptons". In the primary analysis, we excluded patients under the age of 18., and observations where antibiotics or microbial cultures had been ordered within the two weeks prior to the presentation to the emergency department.
Replication	Analysis was replicated on an external site (Boston cohort) using data available through Physionet.
Randomization	No randomization
Blinding	No Blinding

Reporting for specific materials, systems and methods

We require information from authors about some types of materials, experimental systems and methods used in many studies. Here, indicate whether each material, system or method listed is relevant to your study. If you are not sure if a list item applies to your research, read the appropriate section before selecting a response.

Materials & experimental systems

n/a	Involved in the study	
☒	☐	Antibodies
☒	☐	Eukaryotic cell lines
☒	☐	Palaeontology and archaeology
☒	☐	Animals and other organisms
☒	☐	Human research participants
☐	☒	Clinical data
☒	☐	Dual use research of concern

Methods

n/a	Involved in the study	
☒	☐	ChIP-seq
☒	☐	Flow cytometry
☒	☐	MRI-based neuroimaging

Clinical data

Policy information about clinical studies

All manuscripts should comply with the ICMJE guidelines for publication of clinical research and a completed CONSORT checklist must be included with all submissions.

Clinical trial registration	NA
Study protocol	NA
Data collection	See STARR [https://starr.stanford.edu/] and Physionet [https://physionet.org/content/antimicrobial-resistance-uti/1.0.0/]
Outcomes	Microbial culture results, infection coverage rates.
